# The Expression of Vasoactive Intestinal Peptide Receptor 1 Is Negatively Modulated by MicroRNA 525-5p

**DOI:** 10.1371/journal.pone.0012067

**Published:** 2010-08-10

**Authors:** Elisa Cocco, Fabiana Paladini, Giuseppe Macino, Valerio Fulci, Maria Teresa Fiorillo, Rosa Sorrentino

**Affiliations:** 1 Department of Biology and Biotechnology “Charles Darwin”, Sapienza University, Rome, Italy; 2 Department of Cellular Biotechnology and Hematology, Sapienza University, Rome, Italy; University of Georgia, United States of America

## Abstract

**Background:**

The human Vasoactive Intestinal Peptide (VIP) is a neurokine with effects on the immune system where it is involved in promoting tolerance. In this context, one of its receptors, VPAC1, has been found to be down-modulated in cells of the immune network in response to activating stimuli. In particular, the bacterial liposaccaride (LPS), a strong activator of the innate immune system, induces a rapid decrease of VPAC1 expression in monocytes and this event correlates with polymorphisms in the 3′-UTR of the gene.

**Methodology/Principal Findings:**

MicroRNA 525-5p, having as putative target the 3′-UTR region of VPAC1, has been analysed for its expression in monocytes and for its role in down-modulating VPAC1 expression. We report here that miR-525-5p is promptly up-regulated in LPS-treated monocytes. This microRNA, when co-transfected in 293T cells together with a construct containing the 3′-UTR of the VPAC1 gene, significantly reduced the luciferase activity in a standard expression assay. The U937 cell line as well as primary monocytes enforced to express miR-525-5p, both down-modulate VPAC1 expression at similar extent.

**Conclusions/Significance:**

Our results show that the response to an inflammatory stimulus elicits in monocytes a rapid increase of miR-525-5p that targets a signaling pathway involved in the control of the immune homeostasis.

## Introduction

The human Vasoactive Intestinal Peptide (VIP) is expressed and secreted by neurones innervating primary and secondary immune organs, and is involved in smooth muscle relaxation, exocrine and endocrine secretion, and water and ion flux in lung and intestinal epithelia [1]–[4]. VIP has also a strong anti-inflammatory effects in several models of chronic and immune-mediated inflammatory diseases [5]–[10]. VIP signals through three type II, G-coupled receptors, PAC1, VPAC1 and VPAC2, triggering a cascade of intracellular events that differ depending on cell and receptor types [11]. VPAC1 is ubiquitous and highly conserved through species [12]. The down-modulation of the VPAC1 has been described in response to activating stimuli in cells of the immune system [13]. This has been interpreted as a transient switching off of the regulatory pathway mediated by VIP that counterbalances the inflammatory signals. Indeed VIP can modulate the production of some inflammatory cytokines and chemokines and therefore acts as an important player in orchestrating the inflammatory response [14], [15]. Furthermore, VIP has been shown to induce human tolerogenic DCs that, in turn, promote regulatory T cells [16], [17]. Therefore, VIP signalling might play a role in dysregulating the immune system leading to autoimmune diseases. Accordingly, a deficient expression of one of its receptors, VPAC1, has been reported in patients with Rheumatoid Arthritis and this appeared to correlate with polymorphisms at the 3′-UTR of the gene [18]. We have also recently described how LPS treatment can induce a down-modulation of the VPAC1 in monocytes whose kinetics also correlated with variations at 3′-UTR of the gene [19], suggesting a contribution by this region to VPAC1 tuning.

MicroRNAs are a well-established class of small (22 nucleotides) endogenous noncoding RNAs that influence the stability and translation of messenger RNAs. The mature microRNAs are processed from a 70 nucleotide long precursors (pre-miRNA) exported from the nucleus, processed through the action of the cytoplasmic enzyme Dicer, after which the mature miRNA is loaded into the RNA-induced silencing complex (RISC). The RISC complex is guided to the 3′-untranslated complementary region (3′-UTR) of the target RNAs. The matching is imperfect and the so-called “seed region” (2–8 nucleotide) is most important for target recognition and silencing. The recognition of the target sequence can induce inhibition of the translation and destabilization of the target RNA [20]–[23]. More and more reports are involving the activity of microRNAs in the modulation of immune functions as well as in the dysregulation leading to inflammatory, autoimmune diseases [24]–[29]. Having shown that the kinetics of VPAC1 down-regulation in LPS-treated monocytes correlates in particular with SNP rs896 in the 3′-UTR of the VPAC1 gene, we searched for microRNAs having as putative target a sequence that harbors or is close to SNP rs896. MiR-525-5p (MI0003152), mapping in chromosome 19 and showing a sequence partially complementary to a region contiguous to rs896, appeared as the best candidate. We show here that miR-525-5p is upregulated in peripheral blood monocytes upon LPS stimulation and that its enforced expression causes a significant reduction of the VPAC1.

## Results

### MicroRNA-525-5p is predicted to target a region of VPAC1 3′-UTR and it is upregulated in LPS-treated monocytes

**Figure 1:**
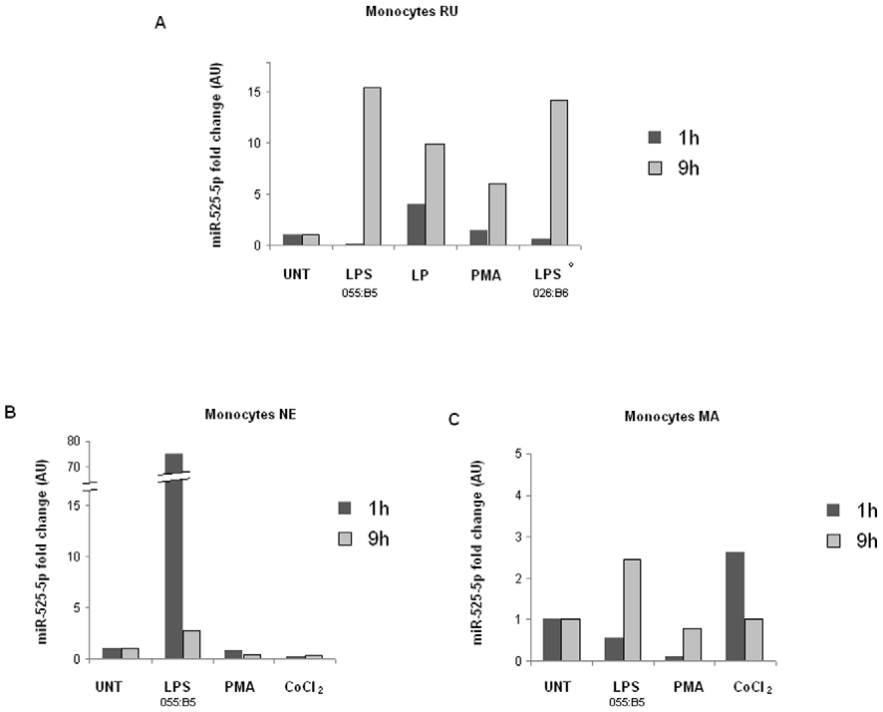
Effect of different stimuli on miR-525-5p expression in human monocytes. Monocytes from three different donors were used to analyze the effect of LPS, lipoprotein, PMA and CoCl_2_ on miR-525-5p expression. (A) Monocytes RU were stimulated with 0.05 µg/ml of LPS from *E coli* 055:B5; 0.05 µg/ml lipoprotein (LP: Pam2CSK4); 5 nM PMA or 0.05 µg/ml of LPS from *E coli* 026:B6 for 1 h or 9 h. Relative gene expression in stimulated cells were compared with untreated cells (UNT), which are set to 1. (B, C) Monocyte NE and MA were stimulated with 0.05 µg/ml LPS (from *Escherichia coli* 055:B), 5 nM PMA and 20 µg/ml CoCl_2_ for 1 h and 9 h. UNT was set to 1.

**Figure 2:**
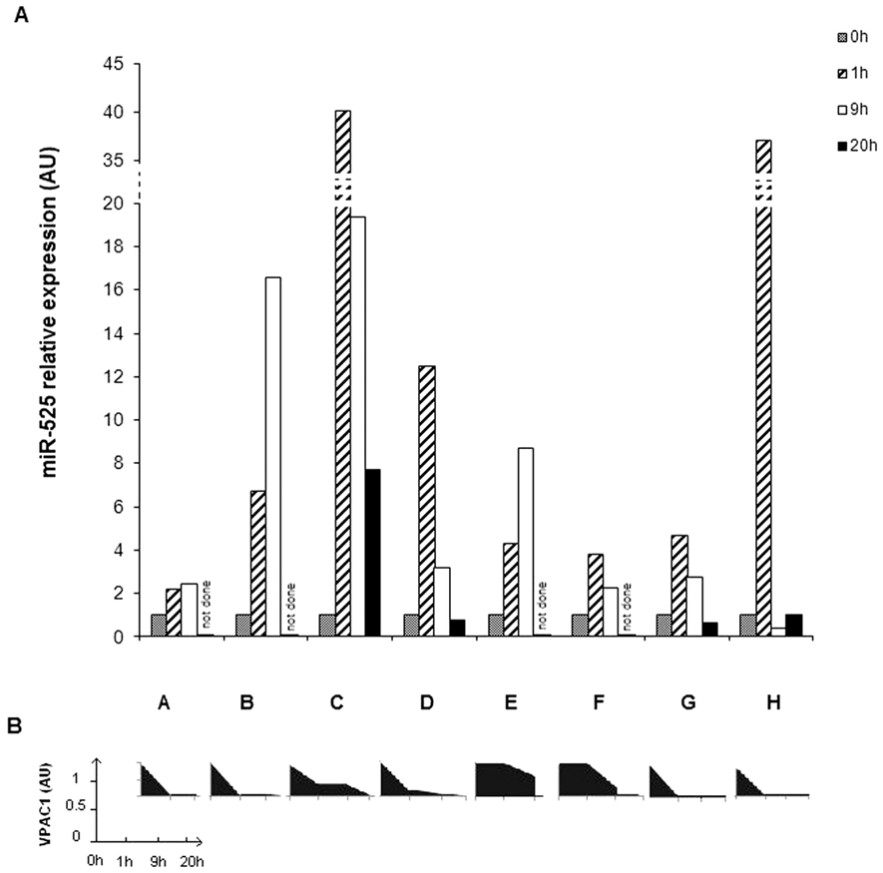
Increased expression of miR-525-5p in LPS-treated monocytes. (A): Relative RT-PCR of miR-525 performed in monocytes purified from 8 subjects (A–H) and unstimulated (0 h) or stimulated with LPS from E coli 055:B (0.05 µg/ml) for 1 h, 9 h and 20 h (only for subjects C, D, G and H). Results were normalized to 18S expression levels. (B): corresponding levels of VPAC1 mRNA for each subject are reported. Results were normalized to GAPDH expression levels. AU: arbitrary units.

According to the observation that SNP rs896 mapping at 3′-UTR of VPAC1 gene correlates with a reduced expression of VPAC1 mRNA and protein in LPS-treated monocytes [19], an on-line search in the miRgen database (http://www.diana.pcbi.upenn.edu/miRGen.html) for microRNAs having as putative target site the region encompassing or close to rs896, was performed. Among the 18 microRNAs putatively targeting the 3′-UTR of VPAC1, miR-525-5p fulfilled this requirement. This prompted us to investigate whether miR-525-5p was expressed in monocytes and whether it was modulated by LPS and/or other stimuli known to activate monocytes. Figure 1 reports the quantitative RT-PCR specific for miR-525-5p following treatment of monocytes from three different individuals with *Escherichia coli* liposaccharide (LPS) from two different serotypes: 055:B5 (0.05 µg/ml) and 026:B6 (0.05 µg/ml); PMA (5 nM), CoCl2 (20 µg/ml) and bacterial lipoprotein (LP) (0.05 µg/ml) after 1 h and 9 h treatment. Interestingly, miR-525-5p was found to be expressed, although at low level, in the untreated monocytes. However, its expression was rapidly upmodulated by LPS treatment. Lipoprotein was also inducing a similar miR-525-5p upregulation (Figure 1, monocytes RU) whereas CoCl_2_ and PMA had no or a negligible effect (Figure 1, monocytes RU, NE and MA). To confirm the effect of LPS on miR525-5p and to further analyze the individual variability shown in Figure 1 in the magnitude of the response to LPS treatment, monocytes from eight additional healthy subjects were also treated with LPS (055:B5 serotype) for 1 h, 9 h, and, when possible, 20 h (Figure 2A). As expected, upon LPS treatment, miR-525-5p expression was promptly induced. The amount and the kinetics of its upregulation varied from one subject to the other: in some cases, the expression increased up to 40 folds (C,H), in some others only few folds (A,F,G); in most of them the highest level was reached already after one hour from LPS addition (C,D,F,G,H), in others the kinetics was slower (B,E). However, in all of them, LPS treatment induced a clear-cut increase in the miR-525-5p intracellular level. In the four cases in which the analysis could be performed at 20 hours time-point, miR-525-5p was reduced towards the background level, indicating a short time range in which its functional effects can be produced. In parallel, the amount of the VPAC1 mRNA was analysed and found to be deeply down-modulated by LPS in all subjects but E and F, in which cases the reduction was less pronounced (Figure 2B).

**Figure 3:**
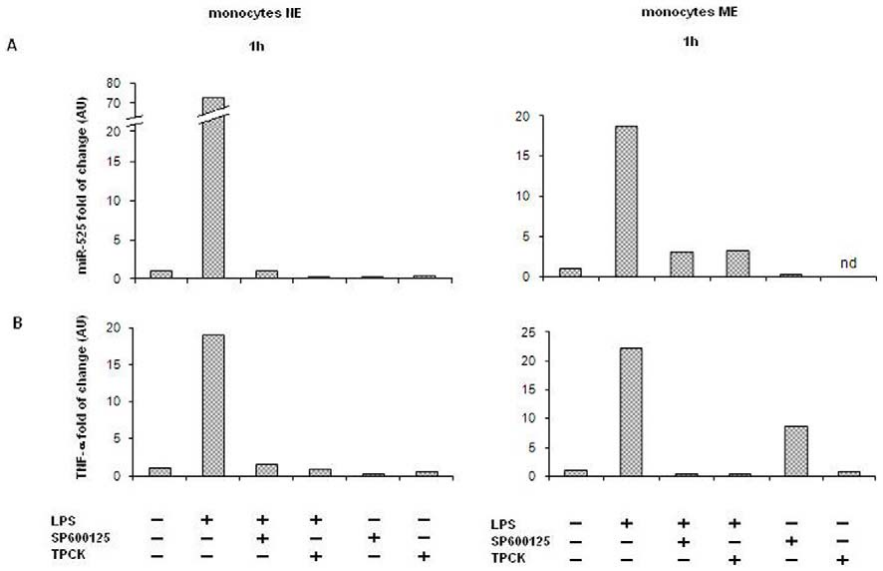
Effect of LPS inhibitors on miR-525 expression in monocytes. Monocytes NE and ME were treated with 0.05 µg/ml of LPS from E. coli 055:B and with 50 µM of SP600125 or 25 µM of TPCK. Total RNA was purified from the respective cell pellets and analyzed by qRT-PCR for the expression of miR-525-5p (A) or TNF-alpha (B). Untreated cells were set to 1. Nd: not done.

It is known that treatment of monocytes with LPS induces a strong inflammatory response involving the NF-kB and the MEK-ERK1/2 pathways and leading to the production of pro-inflammatory cytokines such as TNF-alpha [30]–[31]. To investigate whether these two pathways were also controlling the miR-525-5p upregulation, two inhibitors, TPCK and SP600125, targeting respectively NF-kB [32] and JNK [33], were used in combination with LPS and the expression of miR-525-5p as well as TNF alpha was evaluated in monocytes from two different donors (Figure 3). The data show that, in both cases, TPCK as well as SP600125 inhibitors equally impair the expression of miR-525-5p and TNF-alpha.

### VPAC1 3′-UTR is a target for miR-525-5p

**Figure 4:**
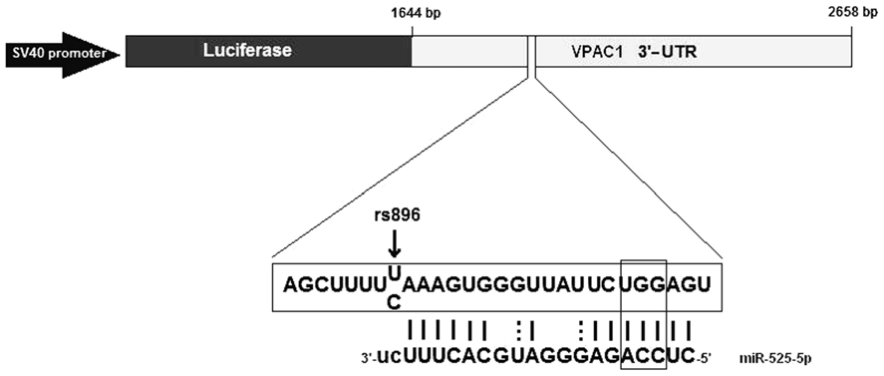
The predicted miR-525-5p target site located in the VPAC1 3′-UTR. Schematic representation of the expression vector pGL3-Promoter containing the VPAC1 3′-UTR. In detail, the target site of miR-525-5p; the arrow indicates SNP rs896 (C/T). The rectangle highlights the mutated bases in the seed region.

**Figure 5:**
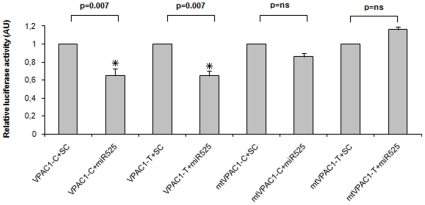
MiR-525-5p inhibits the reporter gene activity. 293T cells were transfected with VPAC1-C and VPAC1-T constructs, harbouring the two alternative 3′-UTR haplotypes or the respective mutated construct and 40 µM of mimic 525-5p RNA or 40 µM of the mimic negative control (SC). Twenty-four hours post transfection, cell were assayed for Firefly luciferase activity and normalized to Renilla luciferase activity, relative luciferase activity (AU). Co-transfection of miR525-5p and the two constructs (histograms 1,2 and 3,4) induced the same degree of inhibition of the reporter activity (35%) (* p = 0.007) whereas the mutated constructs showed no significant effect (histograms 5–8). Scrambled-control (SC) level of luciferase activity was set to 1. Results reported here are the mean±SD of four independent experiments.

The induction of miR-525-5p expression in LPS-treated monocytes, prompted us to verify whether the VPAC1 3′-UTR could be indeed a target for miR-525-5p. A reporter construct was then generated in the vector pGL3 that contains the SV40 promoter driving the expression of a mRNA encoding the firefly luciferase (Figure 4). Two constructs of 3-'UTR of VPAC1 (carrying the haplotypes containing C or T at SNP rs896, named respectively VPAC1-C and VPAC1-T) were cloned downstream the luciferase gene and transfected into 293T cells together with mimic hsa-miR-525-5p or negative-control mimic and pRL-TK to normalize transfection. As further control, a reporter construct with a three nucleotide mutation in the predicted seed region in the VPAC1 3′-UTR was also generated (Figure 4). Twenty-four hours after transfection, the cells were harvested and assayed for luciferase expression. For both VPAC1 constructs, a comparable repression of luciferase activity ranging around 35%, was observed in cells transfected with miR-525-5p compared to those transfected with the negative control (scrambled) (Figure 5, histograms 1 vs 2 and 3 vs 4; p = 0.007). The mutation clearly abolished the effect of miR-525-5p in down-modulating the luciferase activity (Figure 5, histograms 5–8). The small difference between the mutated constructs VPAC1-C and VPAC1-T was not significant. Taken together, these results indicated that miR-525-5p can interfere with luciferase mRNA translation via direct interaction with the VPAC1 3′-UTR.

### MiR-525-5p affects the expression of VPAC1 protein in U937 cell line as well as in human monocytes

**Figure 6:**
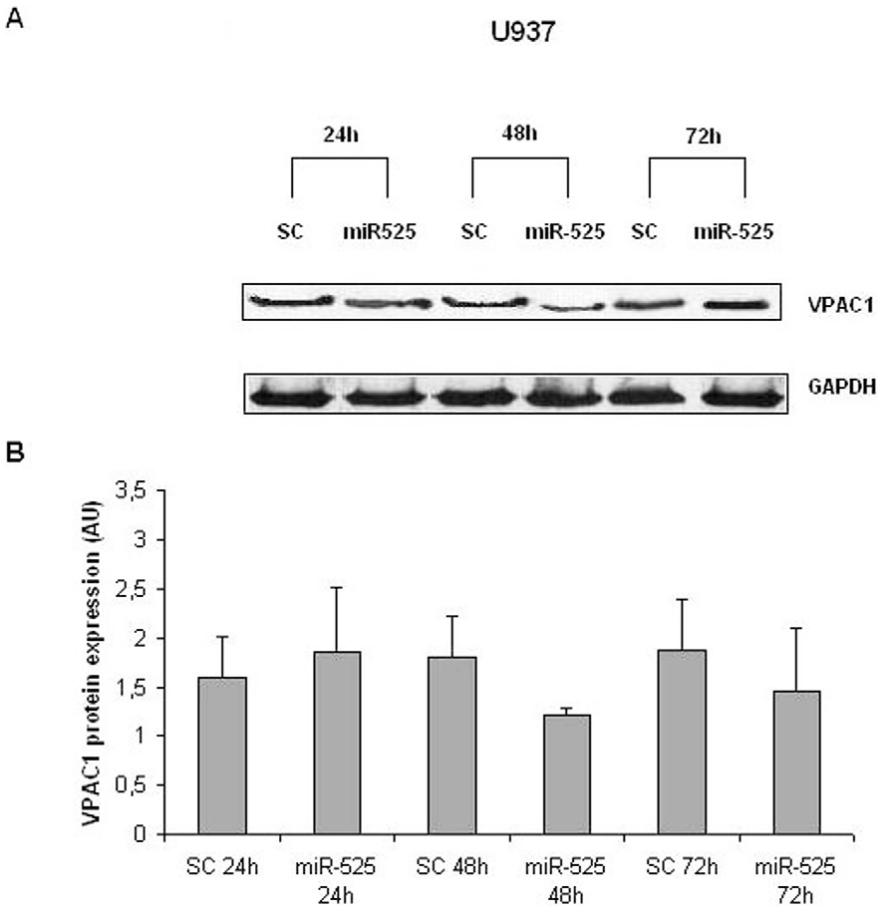
U937 cells line transfected with miR-525-5p decrease VPAC1 protein at 48 h. (A) Western blot analysis of VPAC1 in U937 cells transfected for 24 h, 48 h and 72 h, with 40 µM of miR-525-5p or negative-miR (SC). GAPDH immunoblot was used as loading control. One of three independent experiments is shown. (B) Densitometric analysis: bars represent the mean±SD of three independent experiments. AU: arbitrary units.

**Figure 7:**
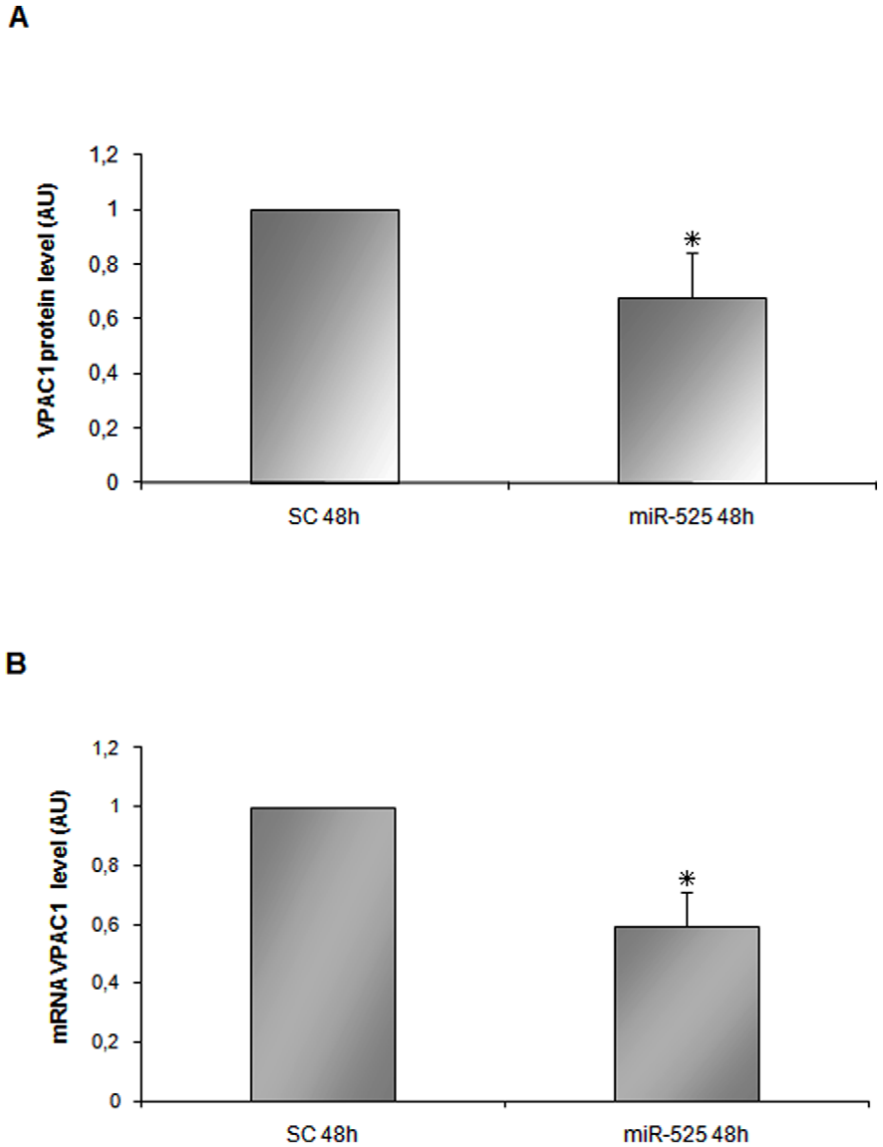
The VPAC1 protein level is decreased in U937 cells after 48 h from miR-525-5p transfection. (A) VPAC1 protein level was evaluated by densitometric analysis of five independent western blots. Bar represents the mean±SD. Scrambled miR was arbitrarily set to 1. VPAC1 protein level was 34% lower in the miR-525-5p transfected cells vs control (* p<0.02). (B) qRT-PCR analysis of VPAC1 mRNA in the same samples as above (*p<0.02). Scrambled-control (SC) level was set to 1. The error bar represents the mean ± SD.

**Figure 8:**
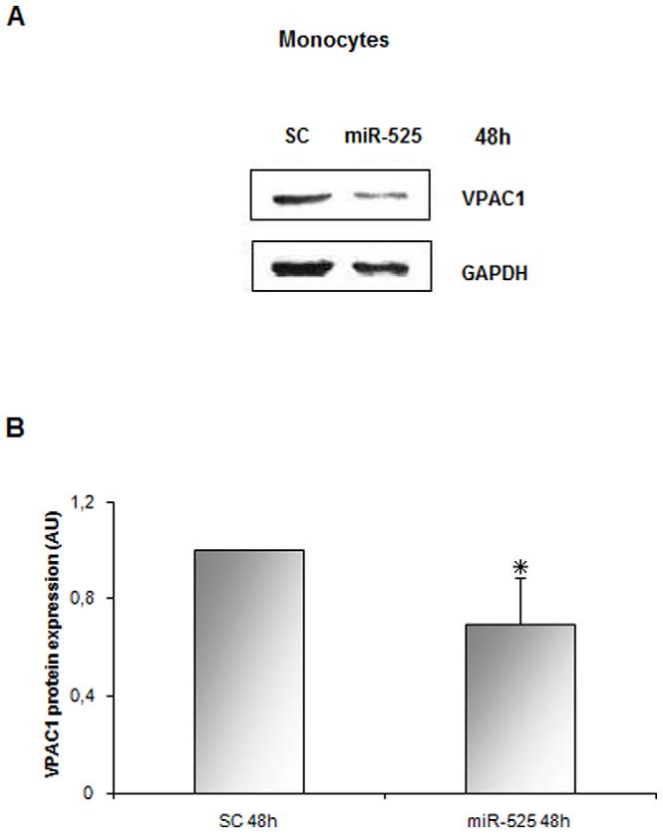
MiR-525-5p induces down-modulation of VPAC1 protein in *ex vivo* transfected monocytes. (A) Western blot analysis of VPAC1 protein in monocytes transfected with miR525-5p and kept in culture for 48 h (representative of seven donors analysed). (B) Densitometric analysis of western blot. Histograms represent VPAC1 protein level as mean±SD in monocytes from 7 healthy donors. The decrease was 31% (*p<0.02). Scrambled-control (SC) was set to 1. AU: arbitrary units.

Each miR can have hundreds of targets and the balance between the amount of that specific miR and the relative abundance of the target mRNAs influences the functional outcome. To verify whether VPAC1 protein expression was indeed regulated by miR-525-5p in a more physiological setting, U937 cell line, in which miR525-5p was not expressed but into which hsa-miR-525-5p was efficiently transfected, were harvested at different times and analysed for VPAC1. Figure 6 shows the results of the western blot analysis. After 24 h, no difference in the level of expression of VPAC1 protein was detectable. However, after 48 h, VPAC1 protein was clearly reduced and after 72 h was still lower than the control. Accordingly, 48 h time point was chosen to statistically evaluate the effect of miR-525-5p enforced expression on VPAC1 in U937 cell line as well as primary monocytes. The experiment was repeated further five times using the U937 cell line (Figure 7A), and a reduction of 34% of VPAC1 protein level was again observed (p<0.02). VPAC1 mRNA level was also affected showing a reduction of about 40% (p<0.02) (Figure 7B). These results, prompted us to investigate the effect of the enforced expression of miR-525-5p in primary monocytes. CD14-positive cells isolated from 7 healthy donors were transiently transfected with miR-525-5p or negative control miR and harvested after 48 h. Although the level of VPAC1 expression in each subject was extremely variable, probably depending on the genetic background or on the level of activation/differentiation of the monocytes, the enforced expression of miR-525-5p led to a consistent reduction of VPAC1 protein compared to the miR control (p<0.02) (Figure 8). This was not paralleled, as in the continuous cell line, by a comparable down-modulation of VPAC1 mRNA, which was variable and not significantly different between monocytes treated with the miR-control or the miR-525-5p (not shown).

## Discussion

Recent research has involved miRNAs in the regulation of innate and adaptative immune responses as well as in the inflammatory networks in various cell and tissue types [29], [34]–[37]. VIP is known to play a relevant role in controlling the immune response through the signalling of its receptors. In particular, VPAC1 gene has been shown to be down-modulated in cells of the immune system after activation [13], [19]. We investigated here whether microRNAs play a role in the LPS-mediated VPAC1 down-modulation in peripheral blood monocytes. Any given microRNA may regulate hundreds of different targets at different spatial-temporal settings [38], [39] and each one needs to be experimentally validated. We focused our studies on miR-525-5p because its target sequence in the VPAC1 mRNA was next to SNP rs896, that was found to correlate with the kinetics of VPAC1 mRNA down-modulation and maps within a stretch of AT (TTTTT/CAAA) where the T/C substitution could modify the secondary structure of the mRNA. Therefore, it was interesting to assess whether SNP rs896, mapping just upstream miR-525-5p target sequence, could influence the binding of this microRNA. In the experimental settings used in this study, we were not able to highlight any significant difference in targeting the two sequences by miR-525-5p. However, there are surprisingly few studies reporting a differential effect of miRNAs targeting polymorphic positions in the 3′-UTR of genes, that usually harbour a considerable number of SNPs [40], [41]. This might be due to technical limitations that do not allow a confident fine tuning of the system in which the ratio microRNA-target is crucial and difficult to quantify in its final combination. Therefore, although we could not see any significant difference in targeting the two sequences by miR-525-5p, this does not exclude that it might be relevant in physiological conditions, especially in those cases in which the induction of miR-525-5p by LPS is less pronounced and/or the competion with other targets is higher. In this context, it is noteworthy that some subjects responded very effectively to LPS increasing miR-525-5p level manyfold whereas some others showed a less dramatic upregulation. Such variation might be due to the genetic background and needs to be further explored since it might influence the individual response to LPS and therefore the subsequent inflammatory cascade. However, a clear cut increase of miR-525-5p was evident in all donors analysed here. The upregulation at the low LPS dose used here was temporary since 20 hours after treatment, the level of miR-525-5p was back or very close to the basal level, suggesting that there is a narrow window during which miR-525-5p may act on its targets. This is in agreement with the hypothesis that one of the tasks of miR-525-5p could be, acting on different targets, to neutralize the negative signals in the presence of an inflammatory input. Consequently, its effect must be timely regulated so that, once the harm stops, the regulatory network can be restored. The relevance and the specificity of the miR-525-5p upregulation in the context of the inflammatory response to bacterial stimulation, is well supported by the observation that only the bacterial products LPS and, at less extent, LP, were able to induce a consistent increase of such miR while other compounds known to activate monocytes such as PMA or CoCl_2_ do not (Figure 1). Both the bacterial compounds are ligands for TLR molecules, LP for TLR2-TLR6 and LPS for TLR4 and their effect is mediated by NF-kB and MAP kinases pathways [42]. Accordingly, specific inhibitors of these two ways clearly inhibited miR-525-5p as well as TNF-alpha upregulation. These data strongly suggest that the upregulation of miR-525-5p is part of a concerted action that orchestrates the monocyte response to the bacterial invasion. The response is effective since the low concentration of LPS (0.05 µg/ml) used to stimulate monocytes induce a strong upregulation of miR-525-5p, suggesting a physiological role for this event. We have shown here that VPAC1, a receptor for a neurokine known to counteract the inflammatory response, is one of the targets for miR-525-5p since its expression is reduced, upon miR-525-5p transfection, both in the U937 cell line and in the peripheral blood monocytes. The co-occurrence of the miR-525-5p induction and of VPAC1 down-modulation already evident few hours after LPS addition [19], together with the data reported here showing that VPAC1 is a target for this microRNA, strongly suggests that the two events co-operate in orchestrating the response of immune cells to a danger signal. It is likely that the effect of LPS on VPAC1 down-modulation is the result of different events, either transcriptional or post-transcriptional, however this is the first report showing how the enforced expression of a microRNA determines its down-tuning. Although there are still several aspects that need to be explored, i.e. why individuals respond so differently to LPS in terms of miR-525-5p induction or whether there are other regulatory mechanisms working in concert with miR-525-5p to tune down VPAC1 and, eventually, how they interact each other, nevertheless the data reported here allow to identify a partnership that is likely to play a role in modulating the native immune response and, therefore, be a potential therapeutic target.

## Materials and Methods

### Bioinformatic prediction of miR target site on VPAC1 gene

The miRgen database (http://www.diana.pcbi.upenn.edu/miRGen/v3/miRGen.html) which integrates analysis from TargetScan, Pictar, and Miranda generated a list of 18 predicted miRNAs targeting the 3′-UTR of VPAC1. Among them, miR-525-5p (GenBank accession no. MI0003152) was chosen fort he position of its putative target sequence near the SNP rs896.

### RNA isolation and miR quantification by RT-PCR Analysis

Total RNA was isolated from monocytes and U937 cell line using the Trizol reagent (Invitrogen, Carlsbad, CA, USA) according to the manufacturer's instructions. RNA quality was monitored by running the aliquots of each sample in 1% agarose gel and by spectrophotometric analysis. Subsequently, 10 ng of total RNA was used to perform reverse transcription using the TaqMan® microRNA assay kit (HSAMIR525 001174, Applied Biosystems, Foster City, CA, USA) and High-Capacity cDNA Reverse Trascription kit (Applied Biosystems) according to the manufacturer's instructions. VPAC1 transcripts were also evaluated using real-time PCR (HS00270351_m1, Applied Biosystems). One microgram of total RNA from each sample was reverse transcribed using random primers of the High Capacity Reverse Transcription kit. Real-time PCR was performed in ABI PRISM 7300 Sequence Detection Systems (Applied Biosystems) using TaqMan®2X Universal Master Mix (catalog no 4324018, Applied Biosystems), in a total volume of 20 µl of reaction mixture. Each sample was assayed in triplicates. The thermal cycling conditions were set up sequentially as follows: denaturating at 95°C for 10 minutes and 60 cycles of 95°C for 15 seconds and 60°C for 1 minute. The fold change of the microRNA and VPAC1 gene in the samples was calculated using the 2^−ΔΔCT^ method. All values were normalized to endogenous control 18S (HS 99999901-S1, Applied Biosystems) for miRNA and GAPDH (HS 99999905, Applied Biosystems) for VPAC1 and were expressed in arbitrary units.

TNF-α mRNA (HS00174128_m1, Applied Biosystems) expression was used to evaluated the effect of the pharmacologic inhibitors on monocytes.

### Plasmids

The pGL3 Promoter Vector, a plasmid that express the Firefly luciferase gene under the control of SV40 promoter and therefore is constitutively expressed, was purchased from Promega (Madison, WI, USA). The pRL-TK, a plasmid that express Renilla Luciferase gene under the control of the HSV-TK promoter, was used as endogenous transfection control.

The human VPAC1 3′-UTR (1307–2790 bp of Genebank accession number NM_004624) was amplified from human cDNA using PCR and the primers: (forward) 5′-gcgcgc tct aga gac act cct aga gaa cgc ag-3′ and (reverse) 5′-gcgcgc tct aga ctc cta tcc aga tga tac atg ag-3′. This 1014 bp fragment was cloned into the XbaI site (underlined in the primers) of pGL3 Promoter Vector downstream the luciferase gene. The mutated construct was generated by using PCR-based mutagenesis of the 1014 bp VPAC1 3′UTR and the primers: 5′-agtgggttattcgtcagtttttgtttggag-3′ and 5′ctccaaacaaaaactgacgaataacccact-3′. This generated a VPAC1-3′UTR with a 3 bp mutation in the predicted miR-525-5p seed region. The resulting fragment was cloned into the XbaI site of pGL3 as above. All constructs were checked by DNA sequencing.

### Cell cultures and stimulation conditions

Monocytes were purified from peripheral blood of anonymous donors from the local data banks using the Monocyte isolation kit (Miltenyi, Bergisch, Gladbach, Germany) according to the manufacturer's instructions. Cells were seeded at concentration of 10^6^ cells/ml in RPMI 1640 supplemented with 10% FCS, 2 mM L-glutamine, 25 U/ml penicillin and 25 U/ml streptomycin (all purchased from Gibco, Invitrogen, Carlsbad, CA, USA) in cell culture plates and treated for 1 h or 9 h with LPS from two different sources: from *E coli* 055:B5 (0.05 µg/ml; Sigma-Aldrich, St Louis, MO, USA) or from *E. coli* 026:B6 (0.05 µg/ml; Sigma-Aldrich) or treated with PMA (5 nM; Sigma-Aldrich) or cobalt chloride (CoCl_2,_ 20 µg/ml; Sigma -Aldrich) or synthetic bacterial lipoprotein (0,05 µg/ml; Pam2CSK4; InvivoGen, San Diego, CA-USA). For treatment with pharmacologic inhibitors, human primary monocytes were incubated for 1 h with SP600125 (50 µM; Calbiochem, Merck KGaA, Darmstadt, Germany) or TPCK (25 µM; Sigma-Aldrich) in the presence or absence of LPS (0.05 µg/ml; from *E. coli* 055:B5, Sigma-Aldrich). 293T cell line (ATCC cat. CRL-11268) was cultured in DMEM medium, supplemented with 10% FCS. U937 monocytic leukemic cell line (ATCC cat. CRL-1593.2) was cultured in RPMI 1640, supplemented with 10% FCS. Cells were maintained in a humidified atmosphere of 7% CO_2_ at 37°C.

### Transfections

The following double-stranded RNAs that mimic mature miRNA, has-miR-525-5p and miRNAs Negative Control were obtained from Dharmacon, (Lafayette, USA). The transfection of 293T cells was optimized utilizing JET PEI Polyplus Transfection Reagent (Polyplus-Transfection, New York, NY, USA). 293T cells were seeded in 24 wells plate and transfected with the luciferase reporter constructs described above (300 ng), pRL-TK control plasmid (5 ng) and the appropriate mimic miRNAs. After 24 h, cells were lysed with Passive Lysis Buffer (Promega) and the luciferase activity was determined. U937 cells line and primary monocytes were transfected with the mimic has-miR-525-5p or the Negative Control using Lipofectamine 2000 Reagent (Invitrogen) according to the manufacturer's instructions. After the indicated time from transfection, RNAs and proteins were extracted for the determination of miR-525 and VPAC1 expression.

### Luciferase assay

Luciferase activity was measured using the Dual-Luciferase Assay kit according to manufacturer's instructions (Promega) with a beta-counter luminometer. Relative luciferase activity was calculated as ratio of the raw Firefly luciferase activity and the Renilla luciferase activity. All assays were performed in triplicate in four independent experiments.

### Protein extraction and Western blot assay

Protein extracts were prepared from monocytes and U937 cell line using RIPA buffer (Sigma-Aldrich), and analysed by SDS-PAGE 12% polyacrilamide gel, blotted on nitrocellulose membrane (GE Healthcare, Piscataway, Nj, USA), and probed o.n. with rabbit polyclonal antibody anti-VPAC1 (kindly provided by Dr. K. Freason). The GAPDH signal was used as loading control (Santa Cruz Biotecnology, Santa Cruz, CA).

### Statistical analysis

All data were expressed as mean±SD, the pair comparison was made, and statistical significance was determined using t-test. Statistical significance was defined as p<0.05.
